# Biologic Stress, Oxidative Stress, and Resistance to Drugs: What Is Hidden Behind

**DOI:** 10.3390/molecules22020307

**Published:** 2017-02-17

**Authors:** Maria Pantelidou, Karyofyllis Tsiakitzis, Eleni A. Rekka, Panos N. Kourounakis

**Affiliations:** 1 Department of Pharmacy, School of Health Sciences, Frederick University, Nicosia 1036, Cyprus; hsc.pm@frederick.ac.cy; 2 Department of Pharmaceutical Chemistry, School of Pharmacy, Aristotelian University of Thessaloniki, Thessaloniki 54124, Greece; karyofyllis@hotmail.com (K.T.); rekka@pharm.auth.gr (E.A.R.)

**Keywords:** cyanosteroids, drug metabolism, drug response, drug toxicity, oxidative stress

## Abstract

Stress can be defined as the homeostatic, nonspecific defensive response of the organism to challenges. It is expressed by morphological, biochemical, and functional changes. In this review, we present biological and oxidative stress, as well as their interrelation. In addition to the mediation in biologic stress (central nervous, immune, and hormonal systems) and oxidative stress, the effect of these phenomena on xenobiotic metabolism and drug response is also examined. It is concluded that stress decreases drug response, a result which seems to be mainly attributed to the induction of hepatic drug metabolizing enzymes. A number of mechanisms are presented. Structure-activity studies are also discussed. Vitamin E, as well as two synthetic novel compounds, seem to reduce both oxidative and biological stress and, consequently, influence drug response and metabolism.

## 1. Introduction

Hippocrates, the ancient Greek philosopher occupied with health and disease, taught that illness was not only pain but also toil. It is the fight of the body for resetting the physiologic condition. He also believed in the curative power of nature (*vis medicatrix naturae*) [1]. Hunter supported that an injury is followed by an attempt of the organism to repair it [2]. Although it has been repeatedly rediscovered that illness has not only been a surrender to an attack but also a battle for health, this has not been understood even to this date [3].

Claude Bernard taught one of the most important characteristics of free life: the stability of its internal environment [4]. During evolution, the body has developed adaptive mechanisms for the maintenance of a steady state, which Cannon called homeostasis [5]. Numerous highly specific homeostatic mechanisms have been found to protect against hunger, thirst, hemorrhage and agents that disturb normal body temperature, blood pH, as well as glucose, protein, fat, and calcium levels in blood. During certain behavioral or emergency states, e.g., fear, rage, pain, the stimulation of the sympathetic system causes a catecholamine discharge [3]. Thus, metabolic and cardiovascular changes prepare the body for fight or flight. It has been established that physiologic equilibrium can be maintained in the presence of numerous agents tending to alter one or more of the constituents of the organism, selectively [5]. In addition to the nervous system, hormones affect the specific adaptive responses of the body. Thus, carbohydrate metabolism is controlled by the pancreatic and adrenal hormones and calcium blood levels are regulated by parathyroid hormones and calcitonin [6,7]. In this review, both biological and oxidative stress are discussed, as well as their interrelation and effect on drug response and metabolism.

## 2. Stress

Non-specific adaptive mechanisms have also been developed to combat aggressors [1,6]. Corticoids, released after activation of the stress phenomenon, are decisive factors. Increased corticoid production enhances systemic resistance and inhibits inflammation [8]. Therefore, non-specific adaptive mechanisms can be stimulated by various factors for protection against different pathogens [9] and stress can be defined as the homeostatic, non-specific defensive response of the organism to challenges [1,10]. Stress is mediated via an adaptive regulation of the nervous, immune and endocrine systems, which collaborate and are mutually affected. Stress is also manifested by morphologic, biochemical, and functional alterations, which could be used as markers of stress [11,12]. An account on the way that glucocorticoids act on neuronal mechanisms during stress response is presented by Shirazi et al. [13].

Stress associated morphologic changes involve involution of thymus and spleen, hypertrophy of adrenals, and gastric ulcerations. Biochemical alterations include plasma glucocorticoid (corticosterone in rats) and uropepsinogen increase [1,11,14]. Morphologic changes on the ultrastructural level, include mitochondrial swelling, endoplasmic reticulum fragmentation, and increase of glycogen granules [15,16].

During stress, there is a stimulation of the hypothalamus-pituitary-adrenocortical axis (HPA) and that of catecholaminergic mechanisms [1,3]. The latter occurs almost immediately, the former some minutes later, demonstrating the important role of the central nervous system during stress. Three of Selye’s collaborators and students published an account of his long-lasting contribution to adaptation and related subjects [14]. In this article, they considered the main implications on the first article launching the concept of biological stress, key ideas and problems that occupied Selye.

We have demonstrated the close relationship between biologic and oxidative stress, the disturbance of the balance between oxidative and antioxidant processes in the body, in favor of the former [17]. Stressors like cold (5 °C), food and water deprivation or immobilization of rats produced a severe biologic stress, as it is demonstrated by great involution of thymus, adrenal hypertrophy, as well as a great increase of blood corticosterone and uropepsinogen (Table 1 and Table 2). Selye had established the mentioned changes for stress evaluation [1]. Later, we added uropepsinogen, as a reliable index to assess a biochemical parameter which increases in parallel with corticosterone [11,17].

**Figure 1:**
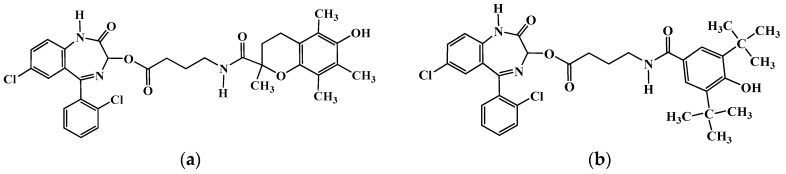
(**a**) Compound I; and (**b**) Compound II.

Having established the biological stress as described, the oxidative stress status remained to be examined. We used α-tocopherol acetate [17]. Furthermore, we designed and synthesized compounds that could combat biologic and oxidative stress [18]. They were composed of a central part, a GABA moiety, esterified with lorazepam, an anxiolytic benzodiazepine, since we have shown that benzodiazepines present some anti-stress properties, but not any antioxidant activity [11]. The amino group of GABA was amidated with carboxylic antioxidants of low toxicity, e.g. trolox (compound I) or 3,5-di-tert-butyl-4-hydroxybenzoic acid (compound II) as shown in Figure 1 [18]. Tocopherol acetate treatment and compounds I and II, reduced biologic stress significantly (Table 2). The characteristic change was that of uropepsinogen, which was reduced by 73% and 76%, respectively, compared with the stressed group. The examined compounds reduced liver oxidative stress by about 30%, as expressed by malondialdehyde determination in the hepatic microsomal fractions [17,18].

## 3. Xenobiotic Metabolism-Resistance to Drugs

Drug metabolism is another adaptive process, developed during evolution [19]. It represents the ability of the organism to resist to changes and keep its internal environment steady [1,4]. Xenobiotics, besides their specific actions, cause the stereotype nonspecific response defined as stress [1,7]. As soon as a biologically active molecule enters the body, it is treated in a number of different ways. Drug biotransformation is one of a number of “ways of loss” [20]. Other ways of loss are excretion [21], deposition in insensitive tissues, e.g., adipose tissue, increase of protein binding, and increased resistance of the blood-brain barrier to permeability [7,22]. Selye studied the effect of hormones, such as ACTH, STH, thyroxin, and a long list of hormonal (e.g., testosterone, estradiol, progesterone), and other steroids, without hormonal or pharmacologic action [1,6]. In many cases, e.g., with PCN, the resistance of the organism was increased tremendously and against a very broad spectrum of toxicants; in other cases, e.g., with progesterone or estrogens, the resistance was increased greatly, but to a certain drug (dicumarol, cocaine) [23,24]. Attention should also be given to the action of the thiosteroid SNL, since it could protect against some heavy metal poisoning, e.g., inorganic mercury as well as dimethylmercury, on top of many organic poisons [25,26]. Concerning the protective effect of SNL treatment against drug poisoning, an interesting observation was presented [27]. It was found that SNL treatment augments production of P-glycoproteins (P-gp) of the liver, a major xenobiotic transporter. The reduction of doxorubicin activity was explained via this mechanism [27]. We found, with Selye, that the increased resistance after treatment with hormones, steroids, or stress was accompanied by reduced blood levels of the toxic agents [24]. This was very clear in many cases, e.g., for zoxazolamine (Table 3, Table 4 and Table 5). The results shown in Table 3, Table 4 and Table 5 clearly demonstrate that treatment with a number of streroids causes a definite increase of the resistance to many toxic agents. In general, the most active was PCN, while SNL was also quite active. Unexpectedly, some steroids, e.g., estradiol, were found to be very protective against selective toxicants, e.g., cocaine [24]. Interestingly, increased resistance was often accompanied by a reduction of the drug plasma levels (observed with PCN and SNL). However, with the potent glucocorticoid triamcinolone, increased resistance to drugs was not accompanied by a great reduction of the drug concentration in blood. 

Increased body resistance to drugs by steroids is mainly due to induction of the hepatic drug metabolism: in order to explain the found increased resistance to drugs after treatment with the above mentioned steroids and other agents, a series of in vitro drug metabolism experiments were conducted, using rat hepatic preparations, after a four day preconditioning with the investigated steroids (Table 6). Table 6 shows the effect of some steroids on drug metabolism, determining zoxazolamine aromatic hydroxylation and ethylmorphine N-demethylation. The most active steroids were PCN again, as well as DEX [22]. This increased metabolic activity goes in parallel with the increased resistance to drugs and the reduced plasma levels. This activity was called “catatoxic” [1,6,22], i.e., an aggressive defensive attack of the body against the invader. Since the resistance of the organism was much higher than the naturally existing mechanisms (homeostasis) by the externally administered steroids (e.g., PCN, SNL, DEX), this phenomenon was called “heterostasis”. Treatment with agents which stimulate physiologic adaptive mechanisms to a higher level establishes a new steady state [29]. The cases of TRIAM and ACTH present interest. They protect, quite efficiently, the body from many drugs, however, as it has been shown [1,21], they neither reduce plasma drug concentrations, nor induce hepatic drug metabolism [23]. This phenomenon has been called “syntoxic activity” [1,30], and is attributed to an increased tissue tolerance to the toxic agent. It could be achieved by different mechanisms, alterations in drug distribution, blood-brain barrier permeability, and in free/protein bound drug [30].

From a study of the effect of various steroids on zoxazolamine distribution in various organs (Table 7), the following could be concluded: zoxazolamine concentration in the brain is higher than that in plasma. This is attributed to the higher lipophilic character of the brain, compared with plasma. This is also true for adipose tissue [30]. Based on the values shown in Table 7, it seems that ACTH treatment has not influenced zoxazolamine concentrations in blood or in the target organ and the offered protection may be due to other reasons. Additionally, ACTH did increase the binding ofzoxazolamine to plasma in vivo and the urinary, as well as the biliary excretion of zoxazolamine, has been increased by 37% after ACTH treatment [31]. After TRIAM treatment, the concentration of zoxazolamine in the plasma of the treated group is somewhat higher than that in the control group, probably because of increased protein-bound zoxazolamine. Furthermore, in spite of the greater presence of the total drug in blood in the treated group with TRIAM, a smaller amount of this seems to reach the brain, related to the control group [30]. This could be attributed to an increased resistance of the blood-brain barrier to zoxazolamine. We also showed that the binding to plasma proteins zoxazolamine was increased with glucocorticoid treatment [30]. In fludrocortisone-treated animals, the concentrations of zoxazolamine in the plasma and brain are reduced, compared with those of the control group, suggesting that fludrocortisone is a weak microsomal enzyme inducer, explaining the moderate protection offered against zoxazolamine intoxication [22]. Additionally, treatment with fludrocortisone seems to reduce the permeability of the blood-brain barrier by zoxazolamine [22]. The concentrations of zoxazolamine in the plasma and brain of PCN treated animals is statistically very much reduced (Table 7). This is attributed to the potent drug metabolising enzyme induction by the cyanosteroid. Moreover, the fraction C_brain_/C_blood_ in the PCN treated animals (2.4) is quite near to the value of the control group (2.7), in the phase of recovery from the pharmacological action. These confirm that the increased resistance to zoxazolamine after the treatment with PCN is due to the induction of the hepatic drug metabolising enzymes and not to an increased tolerance to drugs [22,30].

A wealth of information on the effect of hormones on resistance to drugs and xenobiotic metabolism has been collected by Selye in “Hormones and Resistance” [6]; herein, only an example is indicated (Table 8). From the results shown in Table 8, it can be concluded that ACTH augments drug resistance to zoxazolamine and this could be attributed to both a small induction of drug metabolising hepatic enzymes, but further, and probably mainly, to increased body tolerance to zoxazolamine. These are further supported by the additive actions presented by the combined effects of PCN plus ACTH on both the resistance to zoxazolamine and its metabolism. The opposite happens with STH, which, although it does not affect the resistance of the body to zoxazolamine, and does not alter the metabolic activity of the liver by itself, it does prevent this action of PCN and ACTH [32]. This is not unexpected, because it has been noticed that a mutual antagonism exists between STH and ACTH: the latter inhibits the increase in body weight and stimulation of bone growth elicited by STH [33,34].

Let us keep in mind that stress does not only increase the resistance of the body to demands, among which includes resistance to the presence of xenobiotics, but also, in chronic stress, an important function of the brain may be damaged, leading to cognitive decline and dementia. Activation of the HPA axis in chronic stress and the influence of glucocorticoids in brain cognitive function are presented by Martocchia et al. In that paper, some promising suggestions for strategies against glucocorticoid brain damage are presented [35]. 

## 4. Stress and the Resistance of the Body to Drugs

Further to many cases that Selye has included in [1] concerning the increased resistance to drugs in stressed animals, we have reported that fasting, restraint, hydrocortisone, or reserpine, lead to well-developed stress, resulting in thymus involution (28%–67%), adrenal weight increase (36%–86%), spleen weight decrease (29%–48%) in all cases and plasma corticosterone increase (250%–790%) [21]. Stress induced by the mentioned stressors caused a statistically significant reduction of tetraethylammonium bromide toxicity, expressed as dyskinesia and mortality [21]. This may be explained by the increased urinary excretion of tetraethylammonium bromide which could be attributed to the induction of the corresponding transporter of this drug by corticosterone (phase III in drug metabolism).

According to [21], the produced stress and the accompanying increased resistance to the toxicant were clear. However, the effect that some steroids had on the plasma concentrations, as well as on the drug excreted in urine was only moderately explained. Therefore, a more detailed research on the molecular mechanism of the increased resistance of the stressed animals has been performed. One of the characteristic manifestations of biologic stress is the great increase of corticosterone plasma concentrations in rats. Thus, corticosterone was administered to rats once, 4 h before the experiment, or once per day for three days, as a simulation of the plasma situation in stressed animals, reducing the response to zoxazolamine, methyprylon, and tetraethylammonium bromide from 16% to 39%. Plasma concentrations of zoxazolamine, methyprylon, and tetraethylammonium bromide after the short treatment were slightly reduced (9%–24%) and this has been attributed to the increased hepatic drug metabolic activity, as shown in the in vitro metabolic experiments, using zoxazolamine, ethylmorphine, hexobarbital, and aniline as substrates [9].

From this study, we concluded that: (a) administration of toxic agents in rats leads to elevated corticosterone plasma levels; and (b) administration of corticosterone in rats leads to augmented resistance, connected to decreased plasma levels of the toxic agent, through increased ability of the liver for drug biotransformation. In general, the presence of a toxic agent in the body, in addition to its specific effect, can provoke a non-specific response, stress, which causes HPA stimulation [7,21,36]. Furthermore, we have already shown in this article that many steroids increase the resistance of the organism to drugs, often by induction of microsomal and other enzymes. This route, however, is not the only protective mechanism. It is known that glucocorticoids increase tissue tolerance to toxic agents without an increase of drug biotransformation. This increased tissue tolerance to drugs was called “syntoxic action” [1,6]. The interesting phenomenon here is that one of the stereotype responses to demand—stress—is the increase of plasma corticosterone, as mentioned above. By administration of this natural steroid, the resistance of the body to drugs was increased via hepatic drug metabolising enzyme induction and also through its glucocorticoid properties. This, because tetraethylammonium bromide is not a good substrate of the hepatic microsomal enzymes. Finally, since bioactive compounds like reserpine, hydrocortisone, ether cause stress [21], it could be concluded that drug metabolism, an adaptive process, can be stimulated, in a relatively short time, as a response to need [9]. Some of the abovementioned phenomena concerning resistance to drugs caused by biological stress or steroid hormones related to biological stress have later been rediscovered by others, who, however, presented some valuable therapeutic approaches [37,38]. 

## 5. Biologic Stress and Oxidative Stress

Reports appear occasionally which indicate some relationship between biological and oxidative stress. Thus, intense muscular exercise or fasting cause lipid peroxidation in various tissues [39,40,41]. It is also known that fasting, ethanol, acetone, or diabetes mellitus induce CYP2E1, which is associated with electron leakage, resulting in oxidative stress [42,43,44,45]. In starvation, there is protein synthesis inhibition, cells impairment in expressing genes involved in amino acid import, glutathione biosynthesis, and resistance to oxidative stress [46]. Having this in mind, as well as the great overlapping of pathologic conditions attributed to biological and oxidative stress, the possible relationship between the two was examined. Stress was produced by different stressors, biological and oxidative stress were assessed by the mentioned indices. The influence of alpha-tocopherol was studied and results are shown in Table 9. Both stressor sets caused severe biological stress assessed by objective and sensitive techniques. The use of vitamin E and the novel compounds I and II reduced both kinds of stress significantly. It seems that the two types of stress influence each other and a vicious circle is established. As shown in Table 9, treatment of the stressed animals with alpha-tocopherol brings the values of oxidative stress markers to normal and this is attributed to the antioxidant activity of this vitamin [47]. Results indicate an optimal experimental setup leading to a protective effect of alpha-tocopherol in brain and liver, acting at several subcellular targets of free radical insult, lipids, proteins, and glutathione. Analogous is the behaviour of compounds I and II to both, biological and oxidative, stress. Alpha-tocopherol, in addition to scavenging radicals produced particularly in biomembranes, which induces steroidogenesis [48], may act via protein kinase C inhibition. Activation of the HPA is related to increased protein kinase C activity, and vitamin E, acting as a protein kinase C inhibitor [49] can lead to corticotrophin-releasing factor down regulation [50]. Compounds I and II reduced liver oxidative stress, expressed as malondialdehyde formation, by 34% and 26%, respectively, attributed mainly to potent antioxidant activity. They probably stop the established vicious circle between the two stress phenomena. However, its anti-stress activity via the influence of the HPA may play a considerable role, too [8].

It should be mentioned that other reports have also appeared regarding antioxidant therapy with vitamin E and biological stress. They demonstrated the relationship of oxidative stress with biological stress, the influence of vitamin E on drug metabolism, and resistance to drugs [51,52,53].

## 6. Effect of Stress on Hepatic Drug Metabolic Activity

Selye had suggested, as early as the 1950s, the increased resistance to noxious agents due to stress. Subsequently, it was suggested that stress increased the resistance of the body through elevated hepatic drug metabolic activity [1,6,54,55]. We induced stress in rats by two different methods, assessed the produced stress by objective methods, and used the livers for drug metabolic experiments in order to verify that the elevated resistance to drugs was due to increased hepatic drug metabolic activity, to explain the molecular mechanism of this phenomenon and, furthermore, to examine the effect of an anti-oxidative stress agent, vitamin E [17] and of a novel anti-stress compound that we designed, synthesized, and studied [18]. According to the results, shown in Table 10 and Table 11, it is shown that stress increased hepatic P450 and that drug metabolism in the stressed animals is increased. This is attributed to P450 induction caused by corticosterone [9]. The observed increase in cytochrome P450 content, erythromycin N-demethylation, and 4-nitrophenol hydroxylation shows that the elevated hepatic drug metabolism is primarily due to P450 induction. Although corticosterone is not considered a potent inducer [6,42], it preferably induces CYP3A1, like other steroids [56]. This is further supported by the fact that N-demethylation of erythromycin, a specific CYP3A substrate [57] is greatly augmented under stress conditions [58]. Hydroxylation of 4-nitrophenol is also greatly increased in the stressed animals, and this correlates with the increase in P450 compared with the absolute controls. Treatment with alpha-tocopherol produced no additional effect. The enhanced metabolism of erythromycin and 4-nitrophenol, CYP3A, and CYP2E1 substrates, respectively [57,59], indicate that both isoenzymes are induced in stressful conditions. Treatment of the stressed animals with the novel anti-stress compound I reduced P450 content and N-demethylation of erythromycin by 34% and 40%, respectively, towards normal values. This decrease could be attributed to the limited increase of corticosterone, due to the reduction of biologic stress, centrally from the HPA, as well as indirectly, because of the antioxidant properties of compound I.

## 7. Structure-Activity Relationships

To structurally correlate agents acting on biological stress, oxidative stress, and resistance to drugs, is a difficult task, due to the numerous, often independent, mechanisms involved. Additionally, there are many structures and biological systems implicated [22,60,61].

The case of cyanosteroids, e.g., PCN, is different. Essentially, they possess only one biological activity, CYP induction, and the related response to drugs [1,9]. We have studied the inducing activity on hepatic P450 and, consequently, the following modified resistance to drugs, of many CN-group containing steroids. We distinguished them in three cases: those having the cyano-group at 16α, 16β, and on the plane of the steroidal D ring; the case of the CN-group being at various positions of the steroidal skeleton, as well as having various groups related to CN with varying electronic or steric properties. In an attempt to find the effect of the spatial position (α, β, or on the plane) of the CN-group on drug metabolism and action, we found the following order for their effect on drug metabolism and drug resistance: 16α > CN-in plane > 16β derivatives. This order, as expected, was the same for the resistance to drugs [62]. In our investigation on the effect of the position of the CN-group on the steroidal skeleton on drug metabolism and resistance to drugs [63], we used pregnenolone-2α-CN, 6-CN, 16α-CN, 17α-CN, and 16α-CH_2_CN, studying zoxazolamine paralysis time, digitoxin, and indomethacin mortality, as well as zoxazolamine and ethylmorphine in vitro metabolism. We found that the 16α-CN derivative was the most active in this respect, the 2α-CN and the 17α-CN-pregnenolones were less active, but significantly potent, compared with the controls, while the 6-CN and 16α-CH_2_CN derivatives, as well as pregnenolone itself, were essentially inactive. These differences were explained in terms of an effective or poor fit of the steroids to their receptors. The poor performance of pregnenolone-16α-acetonitrile was attributed to steric and electronic effects. A hypothesis concerning some structural features of the receptor site for its interaction with the cyanosteroid inducers was presented: it seems that there are three fitting areas for the cyanopregnenolones: the 16α-CN, the 3-OH and the lipophilic steroidal skeleton. The most successful and, therefore active, fit is that of the 16α-CN derivative. The second best fit is that of the 2α-CN derivative. If this compound is turned around 180°, it seems to fit the three points fairly well. The fit of 6-CN, 17α-CN-pregnenolones to the described receptor is poorer [63]. Finally, concerning the effect of various groups at 16α-position of pregnenolone with different electronic and steric effects on drug metabolism and response, we studied the following 16α-substituted pregnenolones: CN-, H-, CH_3_-, CN-CH_2_-, -CONH_2_, and -COOH. The conclusions drawn seem to be as follows: Substituent bulk is not important for activity. In this case, the electronic effect seems to be more important, since the most active steroid bears the CN group, which acquires the strongest inductive effect. Thus, short range inductive forces would be expected to hold the substrate in place and determine an effective interaction. In summary, the steroid skeleton can initiate docking to the receptor site, probably through lipophilic interactions; the interaction is completed by a favorable electronic effect at the 16α-position. Regarding the stereochemistry of the 16-substituent, the orientation of the group, but not the bulk, seems to be of importance for a successful and, thus, productive fit [64].

## 8. Conclusions

In this review, biological and oxidative stress were presented from a bio-pharmaco-molecular viewpoint. The mechanisms and interrelations of these phenomena and their effect on drug metabolism remain to be further elucidated. It seems quite reasonable to us, who live in such an adaptable organism as the human body, to perpetually study it, in order to become acquainted with its exquisite structure and function.

## Figures and Tables

**Table 1:** Effect of α-tocopherol on some morphologic indices of stress, uropepsinogen and corticosterone. Stress was caused by cold, food, and water deprivation ^1^.

Treatment	Thymus (mg/100 g)	Adrenals (mg/100 g)	Spleen (mg/100 g)	Liver (mg/100 g)	Body Weight Change (%)	Uropepsinogen (KU/h)	Corticosterone (μg/100 mL)
control	214 ± 9	33 ± 1	251 ± 10	4.70 ± 0.10	4.2 ± 0.1	15 ± 1	20 ± 1
Control + α-Toc	240 ± 10 ** ^++^	39 ± 2 ** ns	281 ± 10 ** ^+++^	4.90 ± 0.10 * ^++^	3.8 ± 0.3 ns ^+++^	15 ± 1 ns ^++^	25 ± 2 ns ^++^
stress	92 ± 7 **	40 ± 1 ***	131 ± 3 ***	3.10 ± 0.02 ***	−28.1 ± 1.0 ***	55 ± 3 **	58 ± 2 **
Stress + α-Τοc	114 ± 5 *** ^+++ xxx^	42 ± 3 ** ns ns	155 ± 5 *** ^+++ xxx^	3.20 ± 0.04 *** ^+ xxx^	−21.0 ± 0.7 *** ^+++ xxx^	21 ± 2 ** ^++^	41 ± 2 ** ^++ xx^
Stress + 48 h rest	142 ± 18 ** ^++^	35 ± 1 ns ^+^	229 ± 11 * ^+++^	3.60 ± 0.20 ** ^++^	−5.2 ± 2.5 *** ^+++^	12 ± 1 ^++^	ns ns

^1^ Table adapted from [17]. Values are mean ± SEM (*n* = 6–10). * *p* < 0.05, ** *p* < 0.01, *** *p* < 0.001 compared to controls; ^+^ *p* < 0.05, ^++^ *p* < 0.01, ^+++^ *p* < 0.001 compared to stress; ^xx^ *p* < 0.01, ^xxx^ *p* < 0.001 compared to α-Τoc, ns: not significant (*p* > 0.05) (Student’s *t*-test).

**Table 2:** Effect of compounds I and II on some morphologic indices of stress and on uropepsinogen. Stress was caused by immobilization, food and water deprivation ^1^.

Treatment	Thymus (mg/100 g)	Adrenals (mg/100 g)	Spleen (mg/100 g)	Liver (mg/100 g)	Body Weight Change (%)	Uropepsinogen (KU/h)
control	214 ± 9	32 ± 0.6	251 ± 10	4.6 ± 0.15	+4.1 ± 0.08	15 ± 0.9
stress	87 ± 9 ***	46 ± 1 ***	127 ± 11 ***	3.4 ± 0.009 ***	−16.8 ± 1.4 ***	127 ± 18 ***
Stress + comp. I	117 ± 22 *** ^+^	34 ± 2 ns ^++^	123 ± 3 *** ns	4.2 ± 0.007 * ^+++^	−10 ± 1.1 *** ^++^	34 ± 8.4 *** ^+++^
Stress + comp. II	151 ± 7 *** ^+++^	40 ± 3 *** ^++^	124 ± 12 *** ns	4.5 ± 0.31 ns ^+^	−3.8 ± 1.6 *** ^+++^	30 ± 13 ** ^+++^

^1^ Table adapted from [18]. Values are mean ± SEM (*n* = 6–10). * *p* < 0.05, ** *p* < 0.01, *** *p* < 0.001 compared to controls; ^+^ *p* < 0.05, ^++^ *p* < 0.01, ^+++^ *p* < 0.001 compared to stress; ns: not significant (*p* > 0.05) (Student’s *t*-test).

**Table 3:** Effect of various steroids on zoxazolamine plasma concentration and action (paralysis time) ^1^.

Steroid (Treatment) ^a^	Zoxazolamine (10 mg/100g b.w., i.p.)			
	Plasma Conc. (μg/mL)		Paralysis Time (min)	
	Treated	Control	Treated	Control
PCN	25.5 ± 0.7 ***	41.4 ± 0.3	19 ± 1 ***	115 ± 17
ESTR	25.3 ± 0.7 ***	30.5 ± 1.1	166 ± 10 ***	203 ± 23
PROG	22.0 ± 1.0 **	27.9 ± 0.7	103 ± 15 ns	100 ± 3
SNL	23.0 ± 1.0 **	26.4 ± 0.9	78 ± 3 **	107 ± 8
DEX	18.9 ± 1.1 ***	28.03 ± 1.7	31 ± 2 ***	128 ± 7
TRIAM	22.0 ± 1.0 **	27.2 ± 1.2	115 ± 9 *	160 ± 15

^1^ Table adapted from [23]. ^a^ PCN: pregnenolone-16α-carbonitrile; ESTR: estradiol; PROG: progesterone; SNL: spironolactone; DEX: dexamethazone; TRIAM: triamcinolone. Treatment: 1 mg/100 g body weight per os (b.w. p.o.), four days. *** *p* < 0.001, ** *p* < 0.01, * *p* < 0.05; ns: not significant (*p* > 0.05) (Student’s *t*-test).

**Table 4:** Effect of various steroids on methyprylon plasma concentration and action (sleeping time) ^1^.

Steroid (Treatment) ^a^	Zoxazolamine (10mg/100 g b.w., i.p.)			
	Plasma Conc. (μg/mL)		Sleeping Time (min)	
	Treated	Control	Treated	Control
PCN	97.3 ± 7 ***	144.5 ± 5.2	14 ± 6 ***	100 ± 15
SNL	80.2 ± 2.3 ***	136.4 ± 13	82 ± 6 **	220 ± 20
TRIAM	131.2 ± 11 ns	119.5 ± 9	68 ± 4 *	101 ± 12

^1^ Table adapted from [28]. ^a^ PCN: pregnenolone-16α-carbonitrile; SNL: spironolactone; TRIAM: triamcinolone. Treatment: 1 mg/100 g b.w. p.o., four days. *** *p* < 0.001; ** *p* < 0.01, * *p* < 0.05; ns: not significant (*p* > 0.05) (Student’s *t*-test).

**Table 5:** Effect of various steroids on tetraethylammonium bromide plasma concentration and toxicity ^1^.

Steroid (Treatment) ^a^	Tetraethylammonium Bromide (10mg/100 g b.w., i.p.)			
	Plasma Conc. (μg/mL)		Dyskinesia (Positive/Total)	Mortality (Positive/Total)
	Treated	Control	Treated	Control
PCN	39.8 ± 2.8 *	45.5 ± 2.3	13/18 *	1/18
SNL	19.4 ± 0.7 **	45.3 ± 2.3	9/18 ***	0/18
TRIAM	24.7 ± 1.6 **	45.1 ± 2.3	3/18 ***	0/18

^1^ Table adapted from [21]. ^a^ PCN: pregnenolone-16α-carbonitrile; SNL: spironolactone; TRIAM: triamcinolone. Treatment: 1 mg/100 g b.w. p.o., four days. *** *p* < 0.001, ** *p* < 0.01, * *p* < 0.05 (Student’s *t*-test).

**Table 6:** Effect of steroids and ACTH on drug metabolism (zoxazolamine and ethylmorphine) ^1^.

Steroid	Zoxazolamine Metabolism		Ethylmorphine Metabolism		Protein ^a^	
	μmol/g/h	% Increase	HCHO μmol/g/h	% Increase	mg/g	% Increase
PCN	42.8 ± 16 *** (16.0 ± 1.4)	166	388.4 ± 22.7 *** (72.4 ± 2.3)	437	104.2 ± 9.1 * (97.4 ± 2.0)	7
SNL	45.7 ± 0.3 *** (19.8 ± 0.6)	130	203.7 ± 13.0 *** (48.3 ± 20.6)	132	99.7 ± 1.2 (97.4 ± 2.0)	2
DEX	65.3 ± 3.0 *** (20.9 ± 2.0)	213	483.0 ± 20.6 *** (68.6 ± 2.3)	603	82.8 ± 1.3 (84.3 ± 1.3)	−2
BET	73.0 ± 1.5 *** (28.3 ± 2.1)	198	422.5 ± 28.0 *** (116.8 ± 13.2)	262	79.4 ± 2.4 (84.3 ± 1.3)	−6
FLUDR	24.8 ± 1.1 *** (16.0 ± 1.4)	54	180.7 ± 17.0 *** (78.0 ± 0.6)	131	77.7 ± 1.6 (84.3 ± 1.4)	−8
TRIAM	28.3 ± 2.1 (24.5 ± 2.2)	15	151.2 ± 13.3 (116.8 ± 13.1)	30	84.3 ± 1.7 (84.1 ± 1.4)	1
ACTH	30.9 ± 1.4 (25.8 ± 1.7)	19	132.3 ± 9.1 (166.0 ± 10.9)	-20	82.7 ± 1.7 (86.0 ± 2.0)	−4

^1^ Table adapted from [22]. ^a^ 9000× *g* supernatant. PCN: pregnenolone-16α-carbonitrile; SNL: spironolactone; DEX: dexamethazone; BET: betamethazone; FLUDR: fludrocortizone; TRIAM: triamcinolone. Numbers in parentheses correspond to controls, which received the liquid vehicle. *** *p* < 0.001, * *p* < 0.05 compared to controls (Student’s *t-*test).

**Table 7:** Effect of steroids and ACTH on the distribution of zoxazolamine in blood, brain and adipose tissue ^1^.

Pretreatment	Group	Zoxazolamine Concentration (μg/mL)			C_br_/C_bl_
		Plasma	Brain	Adipose Tissue	
PCN	control pretreated	35.4 ± 1.1 26.1 ± 1.4 ***	98.2 ± 6.9 63.6 ± 4.8 ***	92.9 ± 3.1 59.1 ± 2.9 ***	2.8 2.4
TRIAM	control pretreated	28.8 ± 1.3 32.9 ± 1.7 *	91.2 ± 4.1 85.4 ± 4.2 ns	81.6 ± 4.1 79.1 ± 2.4 ns	3.2 2.6
FLUDR	control pretreated	26.6 ± 1.4 23.1 ± 1.3 ***	150.6 ± 3.6 101.3 ± 2.3 ***	146.5 ± 7.5 87.8 ± 4.4 ***	5.7 4.4
ACTH	control pretreated	36.9 ± 2.3 37.3 ± 2.1 ns	111.9 ± 7.5 106.7 ± 4.3 ns	106.0 ± 6.5 94.7 ± 3.9 ns	3.0 2.9

^1^ Table adapted from [30]. Each group was composed of 6–13 rats. PCN: pregnenolone-16α-carbonitrile; TRIAM: triamcinolone; FLUDR: fludrocortizone. *** *p* < 0.001, * *p* < 0.05 compared to controls; ns: not significant (*p* > 0.05) (Student’s *t*-test).

**Table 8:** Effect of ACTH, pregnenolone-16α-carbonitrile (PCN) and STH on zoxazolamine paralysis and in vitro drug metabolism ^1^.

Group	Pretreatment	Paralysis Time min	Zoxazolamine Metabolism μmol/g Liver/h (%)	Protein mg/g Liver	Liver Weight g/100 g b.w.
1	control (none)	188 ± 10	2.18 ± 0.12 (100)	93.42 ± 0.66	4.7 ± 0.16
2	ACTH	89 ± 111 ***	2.47 ± 0.18 ns (113)	90.76 ± 1.76 ns	5.41 ± 0.08 **
3	PCN (1 mg)	70 ± 9 ***	4.77 ± 0.17 *** (218)	93.36 ± 1.41 ns	5.21 ± 0.08 *
4	PCN (0.1 mg)	155 ± 12 *	2.90 ± 0.22 * (133)	92.16 ± 1.89 ns	4.94 ± 0.06 ns
5	STH (twice)	184 ± 12 ns	1.79 ± 0.16 ns (82)	105.52 ± 1.56 *	4.58 ± 0.08 ns
6	PCN + ACTH	45 ± 6 *** (***^2^) (*^3^)	5.19 ± 0.16 *** (237)	90.46 ± 0.97ns	6.22 ± 0.08 *** (***^3^)
7	PCN + STH	238 ± 16 ***^4^	2.56 ± 1.12 ** (117)	94.92 ± 2.61ns	4.84 ± 0.19 ns

^1^ Table adapted from [32]. *** *p* < 0.005, ** *p* < 0.02, * *p* < 0.05; ns: not significant (*p* > 0.05) (Student’s *t*-test). Number next to asterisk denotes reference group. Zoxazolamine: 10 mg/100g b.w., i.p.; number of animals in each group: 7–10.

**Table 9:** Effect of α-tocopherol on oxidative stress indices in liver and brain caused by biological stress. ^1^

Treatment	Liver		Brain		
	MDA (nmol/mg Protein)	GSH (nmol/mg Protein)	MDA (nmol/mg Protein)	GSH (nmol/mg Protein)	Protein Carbonyl Content (nmol/mg Protein)
Control	15.9 ± 4.1	2.0 ± 0.4	0.63 ± 0.06	0.27 ± 0.04	0.18 ± 0.01
Control + α-Τοc	2.7 ± 1.1 * ^++^	2.3 ± 0.3 ns ^++^	0.68 ± 0.10 ns ^++^	0.38 ± 0.05 ns ^++^	0.15 ± 0.01 ** ^++^
Stress	49.8 ± 5.8 **	0.4 ± 0.1 **	1.80 ± 0.19 **	0.14 ± 0.02 *	0.28 ± 0.01 **
Stress + α-Τοc	18.8 ± 2.4 ns ^++ x^	1.4 ± 0.1 ns ^++^ ns	0.82 ± 0.13 ns ^++^ ns	0.30 ± 0.02 ns ^++^ ns	0.20 ± 0.02 ns ^++ xx^

^1^ Table adapted from [17]. Values are mean ± SEM (*n* = 6–10). * *p* < 0.05, ** *p* < 0.01 compared to controls, ^++^ *p* < 0.01 compared to stress, ^x^ *p* < 0.05, ^xx^ *p* < 0.01 compared to α-Toc. ns: not significant, *p* > 0.05 (Student’s *t*-test). MDA: malondialdehyde, GSH: glutathione.

**Table 10:** Effect of stress and α-tocopherol on some parameters of drug metabolism ^1^.

Liver Microsomal Parameter	Control Group		Stressed Group	
	Absolute	α-Tocopherol Treated	Untreated	α-Tocopherol Treated
Total P450 nmol/mg prot	0.22	0.30 ** ^++^	0.41 **	0.40 ** ^++^
Erythromycin N-demethylation HCHO nmol/min/nmol P450	0.95	2.20 ** ^+^	1.85 *	2.70 ** ^++^
Nitrocatechol hydroxylation nmol/min/nmol P450	18.5	28.0 ^++^	52.0 **	59.0 ** ^++^

^1^ Table adapted from [18]. Values are mean ± SEM (*n* = 4–6). *p < 0.05, **p < 0.01 compared to control, ^+^ *p* < 0.05, ^++^ *p* < 0.01 compared to stress (Student’s *t*-test).

**Table 11:** Effect of stress and compound I on some parameters of drug metabolism ^1^.

Liver Microsomal Parameter	Control Group	Stressed Group	
		Untreated	Compound I Treated
Total P450 nmol/mg prot	0.23	0.53 **	0.32 ** ^++^
Erythromycin N-demethylation HCHO nmol/min/nmol P450	1.0	2.4 **	1.6 ^+^

^1^ Table adapted from [18]. Values are mean ± SEM (*n* = 4–6). ** *p* < 0.01 compared to control, ^+^ *p* < 0.05, ^++^ *p* < 0.01 compared to stress (Student’s t-test).
